# Sociodemographics and epidemiology of serious infections requiring hospitalization among adults with systemic lupus erythematosus and lupus nephritis, 2000 to 2006

**DOI:** 10.1186/ar4653

**Published:** 2014-09-18

**Authors:** Candace H Feldman, Linda T Hiraki, Wolfgang Winkelmayer, Francisco M Marty, Jessica M Franklin, Daniel H Solomon, Seoyoung C Kim, Karen H Costenbader

**Affiliations:** 1 Brigham and Women's Hospital, Boston, MA, USA; 2 Harvard School of Public Health, Boston, MA, USA; 3 The Hospital for Sick Children, Toronto, ON Canada; 4 Stanford School of Medicine, Stanford, CA, USA

## Background

Serious infections are among the leading causes of hospitalization, morbidity, and mortality in systemic lupus erythematosus (SLE) patients. Patients with lupus nephritis (LN) may be especially vulnerable. We investigated the sociodemographics and epidemiology of serious infections requiring hospitalization in a nationwide cohort of SLE and LN patients enrolled in Medicaid, the US federal-state insurance for low-income individuals.

## Methods

We used the Medicaid Analytic eXtract (MAX) data system, with billing claims and demographics for >24 million Medicaid enrollees from 47 states and Washington, DC, 2000 to 2006. We identified patients aged 18 to 65 years with prevalent SLE (≥3 visits ≥30 days apart with ICD-9 codes of 710.0) and prevalent LN (≥ICD-9 codes for nephritis, proteinuria and/or renal failure on or after SLE diagnosis, ≥30 days apart). We defined serious bacterial, viral, fungal and mycobacterial infections resulting in hospitalization using a validated administrative database method. We stratified infection prevalence in SLE and LN by subtype, sociodemographic factors (age, sex, race/ethnicity, region, socioeconomic status (SES)), and by a validated SLE comorbidity index. We used Poisson regression to calculate incidence rates (cases/person-years) of first and overall infection, stratified by age, sex and race/ethnicity.

## Results

We identified 43,274 patients with SLE and 8,096 with LN. Mean age was 38 (SD 12) for SLE and 34 (SD 12) for LN. In the SLE cohort, 93% were female, 38% were Black, 37% White and 15% Hispanic; and in the LN cohort, 89% were female, 48% were Black, 23% White, and 17% Hispanic. We identified 17,055 episodes of serious infections requiring hospitalization in 7,823 SLE patients (28%) and 7,486 episodes in 3,035 LN patients (38%). Among SLE patients, the highest percentages of infections occurred in 35 to 50 year olds, in females, African Americans, in the South and in the lowest SES group (Table 1). The incidence rates of serious infection per 100 person-years were 15.4 for SLE and 34.5 for LN. In both cohorts, the majority (98%) of infections were bacterial - pneumonia and bacteremia; the most common viral infections were herpes zoster and influenza.

**Table 1:** Serious infections requiring hospitalization in SLE and LN patients, stratified by sociodemographic factors and the SLE comorbidity index

	Prevalent SLE cohort (*n *= 43,274)	Prevalent LN cohort (*n *= 8,096)
Total number of patients with a serious infection, *n *(%)	7,823 (18.1)	3,035 (37.5)
Total number of episodes of serious infections	17,055	7,486
Age, *n *(%)		
18 to 34	2,738 (35)	1,489 (49.1)
35 to 50	3,385 (43.3)	1,063 (35)
51 to 65	1,700 (21.7)	483 (15.9)
Sex, *n *(%)		
Female	7,211 (92.2)	2,684 (88.4)
Male	612 (7.8)	351 (11.6)
Race/ethnicity, *n *(%)		
White	2,613 (33.4)	674 (22.2)
African American	3,465 (44.3)	1,568 (51.7)
Hispanic	915 (11.7)	429 (14.1)
Asian	272 (3.5)	148 (4.9)
Native American	141 (1.8)	59 (5.2)
Other	417 (5.3)	157 (5.2)
Region		
Northeast	1,502 (19.2)	578 (19.0)
South	3,187 (40.7)	1,198 (39.5)
Midwest	1,736 (22.2)	748 (24.7)
West	1,398 (17.9)	511 (16.8)
SES tertile		
SES 1 (lowest)	2,525 (32.3)	937 (30.9)
SES 2	2,402 (30.7)	1,001 (33.0)
SES 3 (highest)	2,441 (31.2)	935 (30.8)
SLE specific risk index^a^		
Index 1 (lowest)	2,298 (29.4)	1,164 (38.4)
Index 2	3,478 (44.5)	864 (28.5)
Index 3 (highest)	2,047 (26.2)	1,007 (33.2)

^a^SLE-specific modification of the Charlson comorbidity index developed by MM Ward and more predictive of in-hospital mortality than the Charlson index among SLE patients.

## Conclusions

In this diverse, nationwide cohort of SLE and LN patients, we observed a significant burden of serious infections requiring hospitalization, most pronounced in LN patients. Further research is necessary to examine risk factors, particularly medication use, by sociodemographic groups.

